# *Toxoplasma gondii* infection in pet cats and their owners in northeastern China:an important public health concern

**DOI:** 10.1186/s12917-021-03110-6

**Published:** 2022-01-03

**Authors:** Xin-Tong Li, Lu Wang, Yuan Ding, Wu-Wen Sun

**Affiliations:** College of Animal Science and Technology, Jilin Agricultural University, Changchun, Jilin Province 130118 People’s Republic of China

**Keywords:** *Toxoplasma gondii*, Pet cats, Owners, Seroprevalence, China

## Abstract

**Background:**

Limited information about *Toxoplasma gondii* infection in pet cats and their owners is available in China.

**Methods:**

In this study, blood samples were randomly collected from 306 pet cats and 397 corresponding pet owners in Jilin province, northeastern China. Sera from the pet cats and the pet owners were tested for anti-*T. gondii* antibodies using an modified agglutination test (MAT) and an enzyme-linked immunosorbent assay (ELISA), respectively. Moreover, the risk factors for *T. gondii* infection in pet cats and corresponding pet owners were explored.

**Result:**

In total, 62 sera out of 306 examined pet cats (20.3%) and 18.1% (72/397) pet cat owners were seropositive for *T. gondii,* respectively. The results of statistical analysis showed that both pet cats and their owners from rural area had significantly higher *T. gondii* seroprevalence than those from urban area (*p* < 0.001). Moreover, owners of pet cas who have the knowledge of zoonotic protozoan diseases had a significantly lower *T. gondii* seroprevalence than those without the knowledge of zoonotic protozoan diseases (*p* < 0.001).

**Conclusions:**

The present results revealed that the seroprevalence of *T. gondii* infection are widespread in pet cats and their owners in Jilin province, northeastern China. Residence area and understanding knowledge of zoonotic protozoan diseases are considered to be raleted to the *T. gondii* infection. Hence, it is necessary to highlight the dangers and protection methods of zoonotic protozoan diseases caused by pet cats, especially in rural area.

## Background

With the rapid development of social economy and living standards, an increasing number of cats and dogs are raised as pets by many families in China [1]. Until now, there are over 50 million pet dogs and 40 million pet cats in China [2]. Considering the huge number of pets in China and the association with their owners, it is necessary for pet owners to know about the information of the zoonotic diseases transmitted by pets [3]. *Toxoplasma gondii* is an important zoonotic parasite belonging to *Phylum Apicomplexa* that is commonly found in warm-blooded vertebrates, including humans and birds [4]. It has been calculated that nearly one-third of the global population has been infected by this parasite [5]. Cats, definitive hosts for this parasite, can discharge oocysts in their feces, resulting in soil contamination with oocysts [5]. Human can infect with this parasite through ingesting raw or undercooked meat containing *T. gondii* cysts, or through ingesting water or food contaminanted with *T. gondii* sporulated oocysts [4, 5].

Many studies have been conducted to explore the potential transmission situation of some zoonotic protozoan diseases between pets and humans all over the world [4, 6, 7]. However, limited information about *T. gondii* infection in pet owners in China can be obtained [8]. Thus, the present study was conducted with the aim to explore the *T. gondii* seroprevalence in pet cats and their owners for the first time in Jilin province, northeastern China.

## Results

In this study, a total of 62 cat sera out of 306 examined pet cats (20.3%) were seropositive for *T. gondii* with titers of 1:25 found in 15 pet cats, 1:50 in 9 pet cats, 1:100 in 9 pet cats, 1:200 in 7 pet cats, 1:400 in 10 pet cats, 1:800 in 9 pet cats, and ≥ 1:1600 in 3 pet cats (Table 1). Considering the species of pet cats, *T. gondii* seroprevalence was ranged from 0 in American Shorthair cat to 26.2% in Chinese Lihua cat (Table 1). *T. gondii* seroprevalence in pet cats from Changchun, Jilin and Liaoyuan were 23.1, 19.8 and 16.9%, respectively. In view of the age of pet cats, the cats were in the 2–3 year old age group have the highest *T. gondii* seroprevalence (22.1%), followed by the ≤1-year old age pet cats (19.8%), and > 3-year old age pet cats (17.6%). Female pet cats (22.3%) had a higher *T. gondii* seroprevalence than male pet cats (18.4%). Moreover, pet cats from rural areas (30.4%) had a significantly higher seroprevalence than those from urban areas (15.2%) (*p* = 0.002).

**Table 1:** Seroprevalence of *T. gondii* infection in pet cats in northeastern China

Variable	Category	No. of sera with MAT titers of							No. tested	No. positive	Prevalence (%)	***P***-value
		1:25	1:50	1:100	1:200	1:400	1:800	≥1:1600				
Species	Chinese Lihua cat	10	4	7	6	7	6	3	164	43	26.2	0.301
	Turkish Angora	2	0	1	0	0	0	0	17	3	17.6	
	Persian cat	0	1	0	0	0	1	0	25	2	8.0	
	Russian Blue cat	0	0	0	1	2	0	0	21	3	14.3	
	American Shorthair cat	0	0	0	0	0	0	0	8	0	0	
	Maine Coon cat	0	1	0	0	1	0	0	13	2	15.4	
	Highland Scottish Fold cat	1	0	0	0	0	0	0	7	1	14.3	
	Siamese cat	2	1	0	0	0	0	0	20	3	15.0	
	British Shorthair Cat	0	2	1	0	0	2	0	31	5	16.1	
Region	Changchun	4	3	4	2	3	6	2	102	24	23.1	0.527
	Jilin	6	3	4	3	5	2	1	121	24	19.8	
	Liaoyuan	5	3	1	2	2	1	0	83	14	16.9	
Age	≤1 year	7	2	2	3	4	3	2	116	23	19.8	0.754
	2–3 year	3	5	5	3	5	5	1	122	27	22.1	
	> 3 year	5	2	2	1	1	1	0	68	12	17.6	
Gender	Male	5	5	6	5	3	5	0	158	29	18.4	0.391
	Female	10	4	3	2	7	4	3	148	33	22.3	
Residence area	Urban	8	7	5	4	3	4	0	204	31	15.2	0.002
	Rural	7	2	4	3	7	5	0	102	31	30.4	
Total		15	9	9	7	10	9	3	306	62	20.3	

The results of testing the *T. gondii* antibodies in the owners of pet cats showed that 18.1% (72/397) owners of pet cats were seropositive for *T. gondii.* Of these, *T. gondii* IgG and IgM antibodies were found in 16.9% (67/397) and 1.3% (5/397) of the owners of pet cats, respectively. With a view to the age of owners of pet cats, *T. gondii* seroprevalence was ranged from 12.6% in the 31- to 40-year old age group to 24.0% in the ≤20-year-old age group (Table 2). The seroprevalence of *T. gondii* infection in owners of pet cats from Changchun, Jilin, and Liaoyuan were 20.8, 18.4, and 13.9%, respectively. Male owners of pet cats (19.4%) had a litter higher seroprevalence than female owners (17.1%). Moreover, owners of pet cats living in rural areas (28.9%) had a significantly higher seroprevalence than those living in urban areas (12.0%) (*p* < 0.001). Owners of pet cats who had the habit of fecal harmless treatment (11.3%), had a lower seroprevalence compared with those who did not have this habit (19.3%), however the difference was not significant (*p* > 0.05). In addition, 39.8% (158/397) owners of pet cats have the knowledge of zoonotic protozoan diseases, in this case, owners of pet cats who understood knowledge of zoonotic protozoan diseases (10.1%) have a significantly lower seroprevalence than those who did not understand knowledge of zoonotic protozoan diseases (23.4%) (*p* < 0.001).

**Table 2:** Seroprevalence of *T. gondii* infection in the owners of pet cats in northeastern China

Variable	Category	No. tested	No. positive	Prevalence (%)	***P***-value
Age	≤ 20 year	50	12	24.0	0.389
	21–30 year	96	19	19.8	
	31–40 year	119	15	12.6	
	41–50 year	80	15	18.8	
	> 50 year	52	11	21.2	
Region	Changchun	144	30	20.8	0.376
	Jilin	152	28	18.4	
	Liaoyuan	101	14	13.9	
Gender	Male	186	36	19.4	0.554
	Female	211	36	17.1	
Residence area	Urban	259	31	12.0	< 0.001
	Rural	142	41	28.9	
Understanding knowledge of Zoonotic protozoan Diseases	Yes	158	16	10.1	< 0.001
	No	239	56	23.4	
Fecal harmless treatment	Yes	62	7	11.3	0.128
	No	335	65	19.4	
Total		397	72	18.1	

## Discussion

In this study, we reported the evidence for the seroprevalence of *T. gondii* infection in pet cats and their owners in Jilin province, northeastern China for the first time. The proportion of *T. gondii* positive in the sera of pet cats and their owners were 20.3% (62/306) and 18.1% (72/397), respectively. The *T. gondii* seroprevalence in pet cats in this study (20.3%) was a median of 20.3% *T. gondii* seroprevalence in cats including stray and pet cats reported by a systematic review and meta-analysis of the seroprevalence of *T. gondii* in cats in mainland China from 1995 to 2016 [9], but a litter lower than 21.67% *T. gondii* seroprevalence in pet cats in Shandong province, eastern China [8].

In China, toxoplasmosis is still an important public health problem because there is an increasing number of AIDS patients, and the number of people living with HIV and AIDS in China is nearly 1,000,000 [10]. It is well known that cats play a crucial role in the transmission of *T. gondii* [4, 5]. In this case, pet cats could be a significantly potential cause of human toxoplasmosis because they frequently intimate contact with their owners. In China, owners of pet cats like to take their pets for a walk in the morning and evening time and the pet cats are free-roaming and might be exposed to *T. gondii* existed in the environment. After this, millions of environmentally-resistant oocysts might be excreted in cat feces, resulting in health risks to animals and humans [5, 11]. Previous studies have showed that the presence of cats at home induces the risk of exposure to *T. gondii* [12, 13]. Thus, it is very necessary to publicize the information of zoonotic protozoan diseases caused by pets to the public. Coincidentally, we found that the owners of pet cats who understood knowledge of zoonotic protozoan diseases (10.1%) have a significantly lower seroprevalence than those who did not understand knowledge of zoonotic protozoan diseases (23.4%) (*p* < 0.001). Therefore, publicity work should be taken to publicize the dangers and protection methods of zoonotic protozoan diseases caused by pets [1]. Furthermore, another known factor that contributes to the maintenance and dissemination of this disease, especially in rural environments, owners of pet cats consuming contaminated vegetables and fruits from home grown and poor sanitary conditions (untreated water). These factors increase the chances of getting infected more than owners of pet cats who live in cities. This is consistent with our research results that owners of pet cats living in rural areas (28.9%) had a significantly higher seroprevalence than those living in urban areas (12.0%) (*p* < 0.001).

Continuing evidence suggests that waterborne transmission of *T. gondii* to humans is common, through the spread of *T. gondii* oocysts by the overland runoff [14]. Thus, fecal harmless treatment of cats can cut down the environmental contamination with *T. gondii* oocysts existed in cat feces [15]. However, owners of pet cats who had the habit of fecal harmless treatment (11.3%), had a lower seroprevalence compared with those without this habit (19.3%) in the present study, however the difference was not significant (*p* > 0.05). Such a large quantity gap of sample-size between owners of pet cats who had the habit of fecal harmless treatment and those without the habit of fecal harmless treatment. Anyway, if we want to cut down the environmental contamination with *T. gondii* oocysts, we must hygienically dispose cat feces in the first place.

## Conclusions

We firstly showed that the seroprevalence of *T. gondii* infection in pet cats and their owners is common in Jilin province, northeastern China. Thus, some control measures should be implemented to reduce *T. gondii* infection in pet cats, and the owner of pets, in the studied regions and elsewhere in China, such as publicity work and fecal harmless treatment.

## Materials and methods

**Fig. 1:**
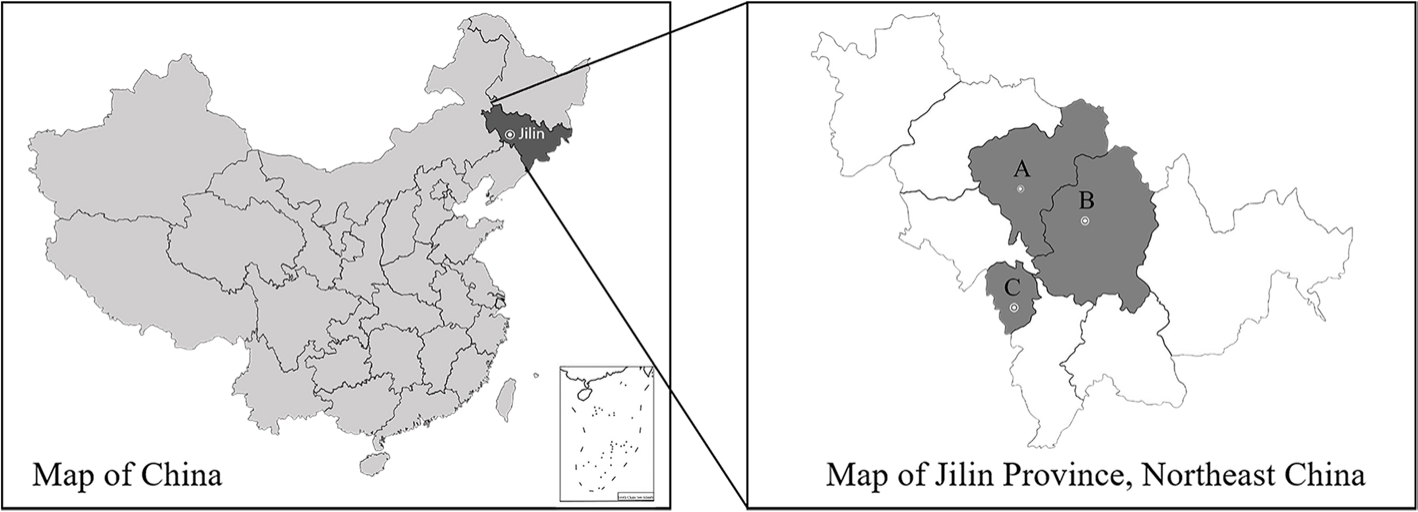
Map showing three cities in Jilin province, northeastern China, where pet cats sampling was performed. A, Changchun; B, Jilin; C, Liaoyuan

Northeast China is an important pet breeding and breeding base in China, and Jilin Province (40°50′ ~ 46°19′ N; 121°38′ ~ 131°19′ E) is located in the central part of Northeast China. Due to geographical advantages and suitable environment, many residents here like to keep cats. The present study was approved by the Animal Ethics Committee of Jilin Agricultural University. From January 2017 to October 2018, 306 pet cats were randomly selected from three regions (Changchun, Jilin, Liaoyuan) in Jilin province (Fig. 1). Questionnaires were provided to cat owners requesting data about each sampled animal. Collected data included information on the species, region, age, gender, and residence area of the pet cat, and their owner’s age, region, gender, residence area, understanding knowledge of zoonotic protozoan diseases and how to deal with feces. Before blood collection of pet cats, a permission was obtained from each pet cat owner and then a local veterinary practitioner was employed to collect the blood samples from the medial saphenous vein of each pet cat. Moreover, we explained the purpose and process of this study to the pet owners and a permission was given to us from the pet owners. After that, nearly 2 ml blood samples were got from the venous blood of each owner by a professional nurse. All blood samples were left about 4 h at 4 °C and then centrifugated at 1500*×*g for 5–10 min to isolate the serum. After isolation, the serum were stored in Eppendorf tubes at − 20 °C before the next step.

In this study, sera of pet cats were tested for anti-*T. gondii* antibodies using a modified agglutination test (MAT) [16, 17]. Slightly, sera were added to the “U” bottom of 96-well microtiter plates, and diluted 2-fold starting from 1:25 to 1:1600. The sera was considered *T. gondii*-positive when the MAT titers ≥25 [10]. Suspicious serum samples were re-tested. Moreover, positive and negative controls were included in all tests. To detect *T. gondii* IgG and IgM antibodies in sera of owners, commercially available enzymelinked immunosorbent assay (ELISA) kits (Haitai Co., Ltd., China) were used following the instructions of the manufacturer. All samples were run in duplicate. SAS version 9.1 was used to do the statistical analysis. *p* value < 0.05 was used to determine statistically significant by a Chi-square test.

## Data Availability

The datasets included in the present study are available from the corresponding author upon request.

## Abbreviations

MAT: Modified Agglutination Test
ELISA: Enzyme-linked Immunosorbent Assay
SAS: Statistical Analysis System
